# Actin—Towards a Deeper Understanding of the Relationship Between Tissue Context, Cellular Function and Tumorigenesis

**DOI:** 10.3390/cancers3044269

**Published:** 2011-12-14

**Authors:** Virginia A. Spencer

**Affiliations:** Cell Culture Essentials, Life Technologies, 7335 Executive Way, Frederick, MD 21703, USA; E-Mail: Virginia.Spencer@lifetech.com; Tel.: +1-240-379-4277; Fax: +1-240-379-4750

**Keywords:** extracellular matrix, laminin, nuclear actin, breast cancer, epithelial

## Abstract

It is well-established that the actin cytoskeleton plays an important role in tumor development yet the contribution made by nuclear actin is ill-defined. In a recent study, nuclear actin was identified as a key mediator through which laminin type III (LN1) acts to control epithelial cell growth. In the breast, epithelial tumors are surrounded by an environment which lacks LN1. These findings point to actin as a potential mediator of tumor development. Here our current understanding of the roles of cytoplasmic and nuclear actin in normal and tumor cell growth is reviewed, relating these functions to cell phenotype in a tissue context.

## 1. Introduction

The cytoskeleton is a filamentous network composed of microfilaments, intermediate filaments and microtubules. Whereas much is known about the function of this structural cytoplasmic network with respect to cell growth and motility, studies performed over the past few decades have shown that actin has a clear presence in the nucleus of normal and malignant cells and an important function in nuclear processes related to cell growth [1]. Recent work has shown that the ECM molecule laminin type III (LN1) influences gene expression in mammary epithelial cells by promoting changes in nuclear actin levels [2]. In light of the observed differences between the microenvironment surrounding a normal and tumor cell, these findings have important implications for advancing our understanding of the genesis and treatment of cancer. Here, our current understanding of the role for cytoplasmic and nuclear actin in normal and tumor cell function is reviewed, with considerable emphasis on breast epithelial cell growth in a tissue context.

## 2. Actin: A Building Block for Cell Structure and Function

Actin is one of the most abundant, evolutionarily-conserved proteins in the cell. It is a member of a superfamily composed of three conventional actin isoforms (α, β, γ) [3] and ten related proteins with 30–70% homology [4-6]. Alpha actin is confined to skeletal muscle cells, beta-actin is present only in non-muscle cells, and gamma actin resides within both muscle and non-muscle cell types. Actin has the capability to dynamically flux between a globular (G-actin) and polymeric form, and, therefore, exists in a multiplicity of structural states throughout both the cytoplasm and the nucleus [7,8]. The assembly of actin into filaments or higher order filamentous networks is dependent on its critical concentration, the presence of ATP and the activity of actin-binding and actin-related proteins [9-11]. In the cytoplasm, as much as 50% of actin is unpolymerized while the remaining population is organized into stress fibers that can be readily labeled with phalloidin, a drug which specifically binds to filamentous actin (F-actin) [12]. These filaments generate force, help form structural scaffolds, act as tracks for the movement of motor proteins and intracellular cargo, and drive cell shape changes that are required for cytokinesis, cell motility and cell adhesion [11].

Over the past few decades, evidence has slowly accumulated suggesting that actin has a distinct presence in the nucleus and is a key player in a diverse set of nuclear functions for many cell types [13,14]. Actin contains a nuclear export sequence but does not contain a classic nuclear localization sequence (NLS) [15]. As a result, it is thought to enter the nucleus either passively through nuclear pore complexes or actively through its interaction with NLS-tagged proteins [15]. *In vitro* cell systems have revealed that nuclear actin binds to and helps mediate the activity of RNA polymerases I, II and III [16-19]. Nuclear actin has been found also to interact directly with different RNA-binding proteins [18,20-22] that function in pre-mRNA processing, as well as mRNA transport, localization, translation and stability [23].

The evidence from these *in vitro* studies has prompted researchers to propose that the functional form of actin in the nucleus is globular. However, the function of cytoplasmic actin is largely dependent on its organization, raising the possibility that the structural organization of nuclear actin plays an important role in its ability to influence transcription and other nuclear processes. Studies on *Xenopus oocytes* have shown that nuclear actin is a component of a highly branched, filamentous structure that spans the length of the nucleoplasm [24,25] and provides mechanical stability to the nucleus [24]. Such a network has not been detected in somatic, eukaryotic cells [26], yet approximately 20% of nuclear actin exists in a polymeric form [8] and many actin-binding proteins which influence the formation of cytoplasmic actin filaments are present in the nucleus [5]. Furthermore, ultrastructural imaging techniques combined with biochemical assays have shown that the nuclei of somatic cells contain a filamentous scaffold that is structurally different from that in the cytoplasm [27,28] but partly composed of actin [27,29,30].

The discrepancy in cytoplasmic and nuclear actin structural organization may result, in part, from their different environments. When compared to the cytoplasm, the nucleus is an extremely compact organelle crowded with approximately two meters of DNA, RNA, and a plethora of protein molecules involved in many nuclear metabolic processes. It is possible that this physical crowding indirectly imposes restrictions on the capacity of actin to form the same type of filamentous structures that can be easily observed in the cytoplasm. The structural differences between cytoplasmic and nuclear actin may also be a consequence of the differential expression patterns for actin-binding and actin-related proteins between these two compartments [4-6].

Although the exact structure(s) of nuclear actin remains to be identified, evidence from several studies shows that nuclear actin can structurally reorganize DNA and nuclear proteins to indirectly promote gene expression. For example, nuclear actin has been shown to be associated with and to be required for the maximal activity of BRG1-containing chromatin remodeling complexes [31]. It has also been shown to be indirectly associated with the p300 histone acetyltransferase through its interactions with hnRNP U [32] and the TIP60 complex [33]. The formation of RNA polII pre-initiation complexes and the physical association of transcription and chromatin remodeling factors with chromatin or the nuclear matrix have been shown to depend on nuclear actin [2,16,31]. As well, actin plays an important role in the directed, long range movement of inducible gene loci to cajal bodies [34] and interchromatin granules [35], regions of the nucleus enriched in RNA processing, RNA metabolism and/or transcription factors [36,37]. This type of long range movement has been shown to require the activity of nuclear myosin [35,38]. Thus, in addition to directly influencing the activity of RNA polymerases, the specific actions of nuclear actin on DNA movement and protein localization make this protein a key factor in the integration of nuclear structure with function.

## 3. The ECM: A Key Factor in Development

The tissue microenvironment is a complex ensemble of ECM molecules, neighboring cells and soluble factors that initiate a carefully orchestrated sequence of interdependent nuclear and cytoplasmic events that guide cell development and function. Importantly, loss of the structure of this environment is considered a hallmark of cancer [39] and believed to be a contributing factor towards tumor development [40].

The majority of adult human cancers originate from epithelial cells [41]. In the mammary gland, there are two types of epithelial cells: myoepithelial cells and luminal epithelial cells. In a healthy individual, the luminal epithelial cells are assembled into a branched network that is surrounded by a layer of basement membrane rich with collagen type IV, and laminin types V and LN1 [42-44]. Contributing significantly to the formation of this basement membrane are myoepithelial cells that produce and deposit LN1 to restrict the growth and maintain the polarity of the luminal epithelial cells [45-47]. Throughout most of a woman's life, the luminal epithelium remains in a state of quiescence surrounded by LN1. However, during pregnancy, the mammary gland ECM undergoes remodeling that allows for the epithelial cells to proliferate and form polarized acinar structures that eventually growth-arrest and produce milk proteins in preparation for lactation [48].

The physical anchoring of LN1 to the cell surface of mammary epithelial cells is mediated by dystroglycan (DG) on the cell surface and thought to recruit the β1-integrin co-receptor that helps mediate LN1 polymerization [49]. Once bound to LN1, epithelial cells initiate a series of intracellular events that favor quiescence and functional differentiation (e.g., milk protein production) [50-52]. Perturbations in DG expression and integrin-LN1 communication have been shown to disrupt these phenotypic outcomes [47,49] indicating that LN1 communication with both receptor molecules is essential for tissue homeostasis.

## 4. Actin—A Key Mediator of ECM-Nucleus Communication

The cytoskeleton relays both extracellular biochemical and mechanical signals to a cell's interior by forming a dynamic yet structural bridge between focal adhesion complexes at cell surface receptors [50,51] and Klarsicht, ANC-1, Syne Homology (KASH)-domain proteins at the outer nuclear envelope [52-54]. The additional transmission of signals to the nuclear interior is further mediated through the interaction of KASH domain proteins with inner nuclear membrane components referred to as Sad1p, UNC-84 (SUN)-domain proteins which, in turn, interact with chromosomes and nuclear components such as lamins [52,53,55]. In addition, actin has been shown to interact directly with nuclear envelope proteins such as lamins A and B [56], which bind to chromatin and transcription factors [57].

Studies from lung, intestinal and breast epithelial cells systems have demonstrated the importance of cytoskeletal actin organization in tissue structure. Inhibition of Rho GTPase-dependent tensional stress on the actin cytoskeleton disturbs the environment surrounding lung epithelial cells, disorganizes the growth patterns of these cells and inhibits their ability to form new terminal buds [58]. In addition, inhibition of Rho-GTPase activity disrupts the polarity of acinar-like breast epithelial cell structures cultured in a LN1-rich gel [59]. The treatment of intestinal epithelial cells with an actin depolymerizing agent has also been shown to play an important role in LN1-mediated intestinal epithelial cell organization into hollow, tubular crypt-like structures [60].

Over the past few decades there have been numerous observations showing that changes in cytoskeletal actin organization influence the cellular localization of proteins that mediate transcriptional events responsible for growth and functional differentiation. The following is a brief account for some of these findings. Culturing mammary luminal epithelial cells in a LN1-rich environment induces changes in the organizational and mechanical properties of cytoskeletal actin that allow for milk protein expression [61]. In human endothelial cells, distortion of the actin cytoskeleton in response to attachment and extension promotes entry into S phase through up-regulation of cyclin D1 levels and down-regulation of cdk inhibitor p27Kip1 levels [62,63]. Chemically-induced disassembly of the actin cytoskeleton prevents signaling proteins from entering into the nuclei of adherent cells and promoting gene expression events that induce growth [62-67]. Interestingly, the state of F-actin assembly in fibroblasts plays an important role in activation of the serum response factor (SRF) [68-70], a transcription factor that works in collaboration with its MAL cofactor to promote the expression of growth factor-regulated immediate-early genes [71,72]. More specifically, G-actin sources which promote MAL nuclear export and prevent its DNA-binding activity to the SRF target gene become depleted in response to serum-induced F-actin formation [70]. Thus, in its globular form, actin serves as a courier protein that regulates the nucleo-cytoplasmic translocation of transcription factors, while, at the same time, communicating to the genome the state of cytoskeletal organization.

Changes in cytoskeletal actin organization also influence steady-state transcriptional activity and the nuclear localization of transcription factors. Disruption of cytoskeletal actin in epithelial cells with latrunculin decreases RNA production [8] and increases the salt solubility of histone acetyltransferases and deacetylases, as well as TAF II 250 and RNA polymerase II [73]. Whether latrunculin targets both cytoplasmic and nuclear polymeric actin to promote these effects remains to be shown.

The roles of actin in both cytoplasmic and nuclear function emphasize the importance of tight regulatory controls over its expression and activity. It is known that actin levels readily increase in response to growth-promoting hormonal factors [74], however, little is known of the *in vivo* controls that restrict its expression. Recent findings indicate that LN1 down-modulates nuclear actin levels in luminal epithelial cells to promote growth arrest [2]. More specifically, exposure to LN1 in culture was shown to dramatically decrease nuclear β-actin levels which, in turn, destabilized the association of RNA polymerases II and III with transcription sites and resulted in a steady-state decrease in both transcription and DNA synthesis [2]. Immunolabeling experiments on tissue sections from pre-pubertal mice showed an enrichment of actin in LN1-depleted regions of the mammary gland that are occupied by actively dividing luminal epithelial cells [2]. Importantly, the effect of LN1 on nuclear actin and its down-stream activities was observed in the presence of insulin, a hormone that promotes DNA synthesis in mammary epithelial cells [75,76]. Thus, LN1 has a dominant growth-inhibitory effect over growth factors [2].

## 5. Actin—A Key Mediator of Tumor Development in a Tissue Context?

The tissue environment is a complex ecosystem composed of a host of both soluble (e.g., growth factors, proteases, cytokines) and insoluble molecules (e.g., ECM molecules) that are secreted or deposited by multiple cell types. The complexity of this milieu combined with the sometimes antagonistic effect of these molecules on cell behavior can make it difficult to appreciate the exact cues that promote and maintain tissue homeostasis in a living organism. However, one thing is certain: tissue architecture plays a critical role in suppressing tumor progression. This concept was born from evidence which showed that introduction of the transforming Rous sarcoma virus (RSV) into chick embryos allows for systemic infection but only transformation when the infected cells were transferred from their tissue environment to an *in vitro* culture [77]. Furthermore, injection of adult chickens with RSV was shown to give rise to tumor development at injured tissue sites [78].

The composition, integrity and physical cross-linking of ECM molecules have a significant impact on the structural properties of a tissue and alterations in these properties can contribute to the malignant phenotype [79]. The ECM associated with breast tumors contains high levels of fibronectin, tenascin, collagen types I, III and IV and proteoglycans [80,81]. Petersen and colleagues have also observed that the cell-stroma interface lining breast carcinomas is either depleted of LN-1 or interrupted with small amounts of discretely concentrated LN-1 foci [46]. Similarly, laminin-5 (LN-5) expression levels have been shown to be markedly down-regulated in breast tumor tissue specimens [82]. The ECM undergoes substantial remodeling through the cleaving activity of extracellular proteinases such as matrix metalloproteases (MMPs) [83]. Increased MMP activity is associated with almost every type of tumor and linked to genomic instability, evasion of apoptosis, angiogenesis, tumor invasion, and metastasis [83,84]. The cross-linking of ECM molecules, an event that is mediated by proteins such as lysyl oxidases, has also been shown to increase tissue stiffness and mediate cancer progression [39,85]. A stiff matrix can cause integrin clustering which enhances ERK activation and increases Rho GTPase-mediated contractility of the cytoskeleton [79,86]. Changes in cytoskeletal tension promote F-actin assembly [86] and there are numerous accounts showing the aberrant expression of Rho GTPases in many tumor types [87].

Alterations in the ECM of tumor tissue only partially explain the discordance in communication between a tumor cell and its extracellular environment. Compared to normal cells, tumor cells express aberrant levels of cell surfaces receptors. For example, the expression of DG has been shown to be frequently down-modulated in human breast, colon and prostate cancers, and inversely correlated with cancer progression [88,89]. Integrin expression also varies between breast epithelial cells exhibiting phenotypic traits that are representative of different stages in cancer progression [47,90]. The β1-integrin is over-expressed in primary and metastatic cancers of the breast, colon, skin and prostate [91-94]. Blocking the binding interaction of this receptor with fibronectin, laminin and collagen causes malignant breast epithelial cells deficient in the assembly of adherens junctions to reverse this phenotype, growth arrest and morph into polarized acinar-like structures with organized adherens junctions [47]. The results of this and previously discussed studies indicate that all three of these outcomes are dependent on cytoskeletal actin. Thus, the act of transformation compromises the structural and functional role for the actin cytoskeleton in both cell-ECM- and ECM-nucleus-communication. Transformation may also alter the mechanisms that influence nuclear actin levels. Indeed, Rao and colleagues have observed that the process of transformation favors a migratory shift of actin into the nuclei of human uroepithelial cells [95]. Although the exact impact of this phenotype on tumor cell behavior remains to be assessed, it is possible that an aberrant increase in nuclear actin levels would have some effect on RNA polymerase function and/or chromatin structure that would mediate gene expression events important for tumor cell development.

## 6. Conclusions

Although a role for nuclear actin in tumor cell growth remains to be established, its involvement is likely when considering the following observations: nuclear actin functions in many aspects of nuclear structure and function [1,13-14], its levels are high in proliferating breast epithelial cells and down-modulated by LN1 [2], and, finally, breast tumors are depleted of LN1 [46] and unable to respond appropriately to homeostatic environmental cues [47]. These findings along with the cross-talk between cytoskeletal actin organization and nuclear actin levels [70] highlight the need for additional investigations into the relationship between cytoskeletal actin, nuclear actin, nuclear function and tumor cell growth within a tissue context in order to develop more effective treatments.
